# Observing flow of He II with unsupervised machine learning

**DOI:** 10.1038/s41598-022-21906-w

**Published:** 2022-11-27

**Authors:** X. Wen, L. McDonald, J. Pierce, W. Guo, M. R. Fitzsimmons

**Affiliations:** 1 Department of Physics and Astronomy, University of Tennessee, Knoxville, TN 37996 USA; 2 Oak Ridge National Laboratory, Oak Ridge, TN 37830 USA; 3 Shull Wollan Center—a Joint Institute for Neutron Sciences, Oak Ridge, TN 37830 USA; 4 Mechanical Engineering Department, FAMU-FSU College of Engineering, Florida State University, Tallahassee, FL 32310 USA; 5 National High Magnetic Field Laboratory, Tallahassee, FL 32310 USA; 6 Present Address: Gloyer-Taylor Laboratories, Inc., Tullahoma, TN USA

**Keywords:** Condensed-matter physics, Fluid dynamics, Techniques and instrumentation

## Abstract

Time dependent observations of point-to-point correlations of the velocity vector field (structure functions) are necessary to model and understand fluid flow around complex objects. Using thermal gradients, we observed fluid flow by recording fluorescence of ${\text{He}}_{2}^{*}$ excimers produced by neutron capture throughout a ~ cm^3^ volume. Because the photon emitted by an excited excimer is unlikely to be recorded by the camera, the techniques of particle tracking (PTV) and particle imaging (PIV) velocimetry cannot be applied to extract information from the fluorescence of individual excimers. Therefore, we applied an unsupervised machine learning algorithm to identify light from ensembles of excimers (clusters) and then tracked the centroids of the clusters using a particle displacement determination algorithm developed for PTV.

## Introduction

Previously, we demonstrated the ability to produce copious ${\text{He}}_{2}^{*}$ excimers in He II using neutron capture, to excite the excimers with lasers and observe the resulting fluorescence^1^. The demonstration was performed in a quiescent environment (i.e., one with very little fluid motion) of an enclosed glass cuvette. Acquisition of such images taken *during flow* around objects is expected to provide data to test theoretical^2^ and computer models that describe the development, intensity, and internal structure of turbulent flow beyond simple systems (*e.g.,* flow around a cylinder or flat plate)^3^. Especially important are data with good spatial resolution of three dimensional (3D) velocity vector fields acquired simultaneously over a field-of-view larger than the object using a technique that minimizes perturbations to flow^4–6^.

Particle tracking velocimetry^7–9^ (PTV) and particle image velocimetry^6,10–13^ (PIV) are well established techniques to observe motion of tracers from which fluid flow can be inferred. In the case of PTV, a relatively small number of tracers are tracked by illuminating the tracers and continuously recording light scattered by typically massive tracers. The tracers follow the fluid flow, so the path of fluid flow close to the tracers can be mapped^14^. In the case of PIV, the field-of-view is flooded with tracers, and the light scattered by the tracers is recorded frame by frame. The velocity vector field is calculated from the cross-correlation of a pair of sequential frames for a user-defined Eulerian grid. The cross-correlation provides the displacement of the tracers within one grid element, which when combined with cross-correlation of other grid elements yields the velocity vector field across the field-of-view.

**Figure 1:**
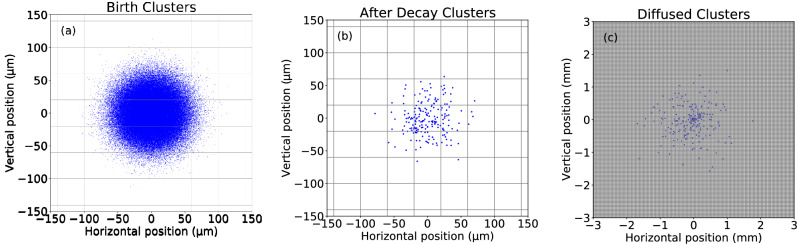
Simulated images of excimer clusters illustrating clusters (**a**) immediately after formation confined to the mean free path of ionizing radiation (**b**) immediately after annihilation of many excimers due to Penning ionization and (**c**) diffusion of excimers after ~ 11 s. The grid represents a camera pixel of 40 µm by 40 µm.

Successful applications of PTV and PIV require continuously (or nearly so) observation of the tracers, and most applications use tracers that do not physically change with time, e.g., because they are solids. Neither condition is satisfied by excimer tracer clusters. Because our experiment records fluorescence of an excimer as a single photon in a small solid angle, and the photon most likely does not enter this solid angle, neither PTV nor PIV are able to extract information about fluid flow directly from individual excimers (discussed later)^11^. In addition, there is no physical mechanism to maintain the shape and size of an excimer cluster. Initially, the density of the excimer cluster is very high (~ 10^15^ cm^-3^) due to the large number of excimers produced within the small region corresponding to the mean free path of the ionizing radiation in liquid He (Fig. 1a)^15^. Soon thereafter, the density decays (10^6–8^ cm^-3^)^16^ through annihilation of excimers due to Penning ionization^17^ (Fig. 1b). The density decays further as the excimers diffuse^16^ in the liquid and the cluster grows (Fig. 1c). Due to the fundamental differences between observation of fluorescence compared to scattered light and the evolution of the size and shape of the excimer cluster, a new approach is required to extract information from transiently visible excimers.

Here, we apply machine learning to identify excimer clusters and use an algorithm for PTV to quantify flow. The technique may facilitate tests of fundamental concepts in turbulence models applied to engineering challenges^18,20^ and enable research in quantum turbulence related to astrophysics^21^ and cosmology^22^. More broadly, the technique to use machine learning to identify clusters and to infer their movement may be applicable to studies beyond fluid flow, e.g., migration of herds of animals^23^, traffic flow^24^ and spread of transmissible diseases^25^.

We demonstrate the ability to track the flow of the normal component of He II across a 1 cm by 1 cm field-of-view. Motion is evident in the images of the fluorescence and by tracking individual excimer clouds identified using an unsupervised machine learning clustering algorithm. We measured the correlation between the velocity flow field and power applied to heaters that create thermal gradients. Evidence for flow is unequivocal. Flow brings excimers from a portion of the cryostat illuminated by the neutron beam into the region of the cryostat that is in the shadow of the neutron beam—a region in which excimers cannot be produced. All other approaches using ${\text{He}}_{2}^{*}$ excimers to track flow, monitor the evolution of fluorescence of ${\text{He}}_{2}^{*}$ excimers produced in a highly confined region^26,27^. Our approach produces ${\text{He}}_{2}^{*}$ excimers throughout a large volume, then we identify clusters of ${\text{He}}_{2}^{*}$ excimers with machine learning and record the displacements of the clusters with time. Provided the clusters do not disperse (quickly), the approach is more akin to PTV than to PIV. The new approach lays the foundation to observe flow that fully encompasses cm-sized objects and provides opportunities to observe point-to-point correlation in space and time of the velocity vector field (dynamic structure functions), albeit, presently lacking the spatial resolution that can be obtained using massive tracers.

## Methods

A detailed description of the experimental setup is given in Ref.^1^ Briefly, an Oxford OptistatCF2 cryostat (a static exchange gas continuous flow cryostat) with quartz windows transparent to infrared light contains a 10 mm $\times$ 10 mm $\times$ 35 mm quartz cuvette (FireflySci). The cuvette was connected to a 3.5 L reservoir filled with a mixture of ^3^He and ^4^He in the ratio of 1 to 1900 (pressure at 300 K is 67 kPa). The cuvette was cooled by the liquid He bath to 1.6 K. The experimental setup for the present experiment (Fig. S1) differs from Ref.^1^ in two ways. First, a 6.4-mm thick plate of boron-nitride (BN) (an effective neutron absorber) was placed on the front surface of the cryostat to block the neutron beam from entering either the top or alternately the bottom half of the glass cuvette to form a neutron shadow. There are no excimers created in the shadow of the neutron beam. Thus, fluorescence observed in the neutron shadow must originate from excimers that moved from the neutron-illuminated region into the shadow. Second, a fiberglass (G-10) rod was inserted into the cuvette but displaced away from the laser sheet by about 1 mm. The rod was wrapped with 40 cm of 32 AWG Nichrome wire as two separate coils 1 cm above and below the center of the camera’s field-of-view (Fig. S1). The coils could be energized independently to produce a thermal gradient. By passing current through a coil, heat was created and transported away from the coil via thermal counterflow: the normal fluid moves at a speed proportional to the heat applied, and the superfluid moves in the opposite direction to ensure no net mass flow^28^.

The experiment required a pulsed laser and two continuous wave lasers to efficiently produce excimer fluorescence. An EKSPLA brand laser produced the pulsed 905 nm laser light with frequency of 1 kHz and pulse duration of 4 ns. The energy of each pulse was 0.9 mJ. Two custom-made 200 mW diode lasers (AdValue Photonics) produced the 1073 and 1099 nm continuous wave light. The light from the three lasers was focused to a cross-section 1 mm wide (along the camera axis) $\times$ 10 mm tall that illuminated the 20 mm length of the cryostat along the axis of the neutron beam.

The experiment also requires a camera to record the fluorescence. We used a Princeton Instruments PI-MAX3 1024i ICCD camera^29^to record images with resolution of 256 $\times$ 256 pixels mapped onto a field-of-view measuring 10 mm $\times$ 10 mm. The field-of-view was between the two heater coils. The camera was equipped with a light intensifier P46 phosphor. The lens of the camera was focused onto the plane illuminated by the lasers. The axis of the camera was perpendicular to this plane and to the neutron and laser beams. Data were exported using the vendor’s LightField software^29^ and analyzed using a Jupyter Notebook platform.

### Excimer production by neutron beams

Neutron capture by one ^3^He atom yields ionizing radiation in the form of a proton and triton with energies of 573 and 191 keV, respectively, and path lengths in liquid He of ~ 59 and ~ 15 μm, respectively^15^. Previously, we measured the mean size of the excimer ensemble (cloud) to be 25(3) μm [schematically shown in Fig. 1a,b]^1^. Protons and tritons create helium ions ${\text{He}}^{ + }$, electrons and excited state $He^{*}$ atoms. One $He^{ + }$ and 0.45 $He^{*}$ are produced^30^ for every 43(1) eV deposited by α and β particles^31,32^, which we assume is the case for tritons and protons in order to estimate the number of excimers produced by the absorption of a neutron. After thermalization, ion–electron pairs in proximity to ground state He atoms can form He dimers: $e^{ - } + {\text{He}}^{ + } + {\text{He}} \to e^{ - } + {\text{He}}_{2}^{ + }$ and then combine to form helium excimer molecules: $e^{ - } + {\text{He}}_{2}^{ + } \to {\text{He}}_{2}^{*}$^33–35^. In the absence of spin correlation between the electron and dimer, which is reasonable for very dense ionization events produced by heavy nuclei, 75% of the ${\text{He}}_{2}^{*}$ should be in the spin triplet state, $a_{3} {\Sigma }_{u}$, and the remainder in the spin single state. ${\text{He}}^{*}$ atoms also create helium excimer molecules: ${\text{He}}^{*} + {\text{He}} \to {\text{He}}_{2}^{*}$^36^. Sato et al.^30^ calculate 17% of the ${\text{He}}^{*}$ occupy the spin triplet state. Because a spin-flip transition is forbidden, 17% of the $He_{2}^{*}$ created from $He^{*}$ in proximity to ground state atoms will be in the spin triplet state. Thus, we expect for every neutron captured *not more than* $\frac{{764\,{\text{keV}}}}{{43\,{\text{eV}}}}\left( {1 \times 0.75 + 0.45 \times 0.17} \right)\sim 14,700$ ${\text{He}}_{2}^{*}$ excimers will be produced *in the* $a_{3} \Sigma_{u}$ *spin triplet state*. On account that so many excimers are created in a small region (764 keV deposited along 74 μm, i.e., ~ 10 keV/µm from neutron capture compared to 40 eV/µm for a 500 keV electron^37,38^), many excimers will be lost due to Penning ionization. The fluorescence captured by the camera will correspond to the product of 15 k excimers, the solid angle subtended by the lens, and the quantum efficiency of the image intensifier (0.36^29^). The product—an upper limit—is ${\mathcal{O}}\left( {10^{2} } \right)$ excimer fluorescence events per laser pulse exceeds our observations (discussed later) by about one-third. We note Penning ionization will reduce the observed value. Nevertheless, the calculated value is reasonably consistent with observation.

## Results

The $a_{3} {\Sigma }_{u}$ spin triplet state can be excited with a pair of 905 nm photons to emit a 640 nm photon (the fluorescence), which can be recorded by a camera^39,40^. The time to emit a photon is 48(2) ns^41^. Because the normal component of He II is viscous, this component drags the excimers with it^42^; thus, the location of the fluorescence as a function of time provides a means to record the motion of the normal component. While excimers are produced by the neutron beam throughout the bottom 1 cm^3^ of the cuvette, only excimers within 1 mm of the plane that bisects the cuvette and is perpendicular to the camera’s axis are illuminated by the lasers and can fluoresce (Fig. S1). This plane is also the focal plane of the camera.

We measured the fluorescence for power ranging from 0 to 30 mW applied either to the top or bottom heater, with the boron-nitride (BN) absorber either blocking the neutron beam from entering either the top or bottom of the cuvette, i.e., four combinations. A movie of the fluorescence recorded by the camera’s field-of-view as a function of time for power of 7.3 mW applied to the bottom heater shows the induced flow (Fig. S2).

**Figure 2:**
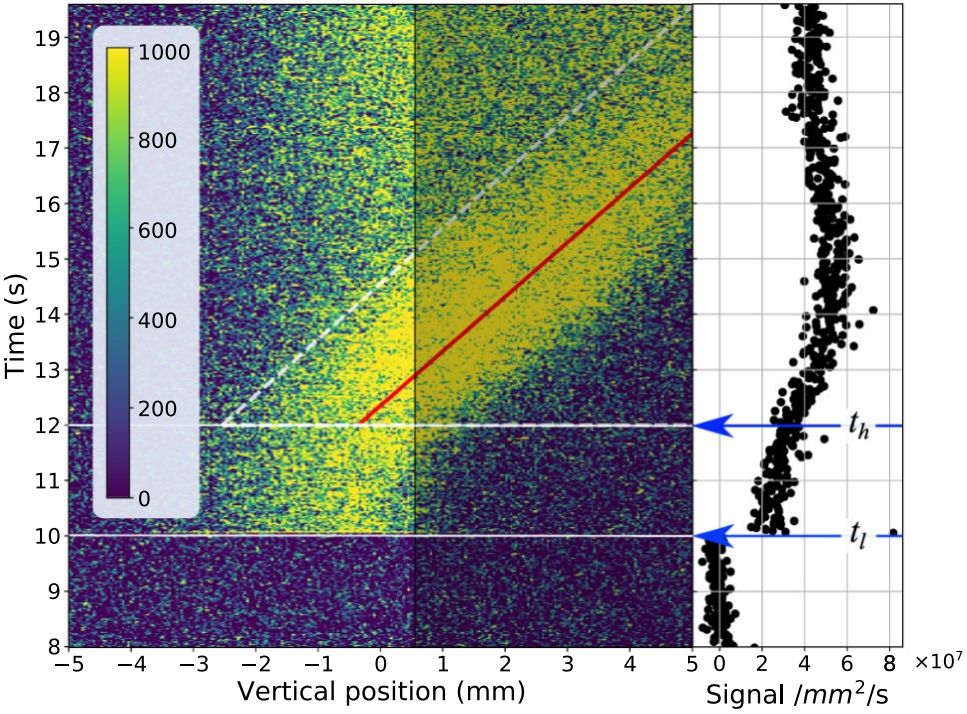
(left) Excimer fluorescence integrated across the horizontal field-of-view per second recorded versus time and vertical position. Only the region for < 0.5 mm is illuminated by the neutron beam. The red line is the best fit to the peak of the fluorescence position vs. time. The inverse of the slope of the red line is the velocity of the flow for a heater power of 7.3 mW (this case). (right) Fluorescence integrated over the entire field-of-view vs. time. The neutron shutter was opened at 5 s, the laser shutter opened at $t_{l} = 10$ s, and the bottom heater energized at $t_{h} = 12$ s.

The fluorescence integrated across the horizontal dimension of the camera (i.e., for each image in the movie Fig. S2) is plotted as a function of vertical position and time in Fig. 2. The region of the cuvette in the neutron shadow corresponds to vertical position > 0.5 mm relative to the center of the field-of-view at 0 mm. The neutron beam was turned on at time = 5 s, and the recording started at time = 8 s. Once the neutron beam was turned on, excimers form in the neutron-illuminated region (laser light enters the cryostat from the side opposite to the BN neutron beam blocker, so only the neutron beam is blocked). The lasers were turned on at $t_{l} = 10$ s, and fluorescence was immediately observed. Fluorescence is absent for vertical position > 0.5 mm for time < 12 s. The heater was turned on at $t_{h} = 12$ s. Shortly thereafter, fluorescence was observed in the neutron shadow, and the fluorescence moved to larger vertical positions with increasing time. Eventually, the field-of-view became saturated with fluorescence [Fig. 2 (right)].

**Figure 3:**
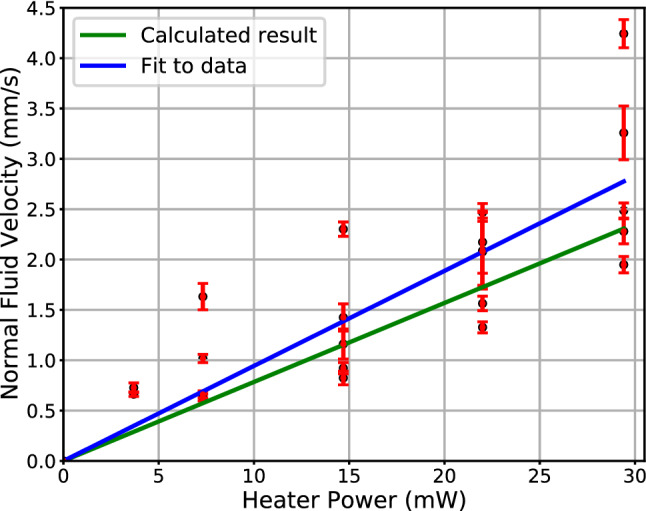
Mean velocity of fluorescence motion (aka fluid flow of the normal component) vs. heater power (Fig. 2). The error bars represent the uncertainty of the fitted velocities. The green line was obtained using Landau and Lifshitz’s equation for heat flux in He II,^44,45^ and the blue line is the best weighted fit to the data with slope of 0.09(1) m/J.

The red line in Fig. 2 represents the peak of the fluorescence that is indicative of flow. To obtain the line, we fitted Gaussian profiles in the direction perpendicular to the line along its length. The fitting optimized parameters of the Gaussians, e.g., width and amplitude, background, and importantly the slope and intercept of the line. The inverse of the slope is the component of the velocity of the normal fluid flow along the vertical direction (corresponding to positive vertical position in Fig. S2). Shown in Fig. 3 is the normal fluid (vertical) velocity as a function of heater power for many measurements. The error bars represent uncertainties of the linear fits (corresponding to “red lines” for each power) to the data. Landau and Lifshitz^43,44^ provide an equation $q = \rho STV$ that relates the heat flux, $q$, to normal fluid velocity, $V$, through the mass density of liquid He,$\rho$, its entropy, $S$, and temperature, $T$. We estimated a value of $q$ as the ratio of the heater power, $P$, to the surface area of the coil of $A = 2.4$ cm^2^. Using values of $\rho = 1.452 \,{\text{g}}/{\text{cm}}^{3}$, $S = 0.236 {\text{J}}/{\text{g}} \cdot {\text{K}}$ for T = 1.55 K^45^, we calculated the expected value of $V$ vs. power $\left( {V = \frac{1}{A\rho ST}P} \right)$, which is shown as the green line in Fig. 3. The blue line is the error-weighted best fit to the data subject to the constraint $V = 0$ for $P = 0.$ Because not all windings of the coil were fully exposed, the effective surface area of the coil in the experiment is less than our estimate of $A$, consequently, the slope [= 0.09(1) m/J] of the fitted line (blue) is somewhat larger than $V\left( P \right)$ (green line).

## Discussion

Electrons produced by electric discharge from needles^41^ or laser induced ionization^46^ are means to initially produce high densities (e.g., $1.4 \times 10^{9}$ cm^-3^ ^40^) of excimers in very localized regions (e.g., ~ 0.005 cm^3^, see Fig. 2 of Ref.^47^), although Penning ionization of the excimers is expected to reduce the density with time. The images of fluorescence in Ref.^47^ are well-defined and typical of all other approaches to track flow using ${\text{He}}_{2}^{*}$ excimers. In contrast, the images Fig. S2 are not well-defined. The lack of definition is due to two challenges: (1) the small number of neutrons captured (and excimers produced throughout a cm^3^ volume) and (2) the low probability 1/42 that a photon emitted by an excited excimer travels in the solid angle of 0.3 sr subtended by the camera lens.

Because neutron capture can occur anywhere in the unshadowed part of the 1 cm^3^ volume of the cuvette, and the neutron capture rate is low, the data are sparse. The neutron capture rate, $R = 22\left( 4 \right){\text{n}}/{\text{s}} \cdot {\text{cm}}^{3}$, is the product of the neutron beam intensity, $1.3\left( 2 \right) \times 10^{5} {\text{n}}/{\text{s}} \cdot {\text{cm}}^{2}$ integrated over neutron wavelength from 3.07 to 5.53 Å, the average neutron cross-section of ^3^He $\overline{\sigma } = 13511$ barns for this wavelength range, and the density of ^3^He, $1.26\left( 8 \right) \times 10^{16} {\text{cm}}^{ - 3}$. Using the upper limit of 14,700 (spin triplet state) excimers per neutron capture, and $R$, excimers are produced at a rate of $\sim 10^{5} {\text{s}}^{ - 1} {\text{cm}}^{ - 3}$. This value corresponds to an excimer density averaged over the volume of liquid illuminated by the neutron beam. The neutron capture rate can be increased by either increasing the neutron beam intensity (e.g., by performing the experiment at a different neutron source) or by increasing the concentration of ^3^He. An increase in the ^3^He concentration will (1) increase the possibility that the ^3^He alters the hydrodynamics of the ^4^He liquid, and (2) lead to highly non-uniform absorption of the neutron beam across the field-of-view. Our choice of the ^3^He:^4^He ratio balances the need for many excimers and the consequences of too much ^3^He.

Shown in Fig. S3 are images of sequential frames for which excimer fluorescence was greater than four times the standard deviation of a background measurement (a measurement with the local neutron shutter closed so no excimers were produced), > 4σ. Such excimer fluorescence is called an excimer peak. Each frame contains ~ 80 peaks (or records of 80 photons) over the 1 cm^2^ view. An entire series of 1000 such frames is shown in a movie, Fig. S4 for one experiment (heater power 7.3 mW). Because the trajectory of an emitted photon is random, there is only a 1 in 42 chance that an excimer producing a photon recorded in the second image [Fig. S3 (right)] is correlated with the same excimer that produced the photon recorded in the first frame [Fig. S3 (left)], thus, the cross-correlation of the two images is unlikely to yield a significant result. To quantify how unlikely, we simulated random configurations of tracers of varying number imposing known displacements of the second configuration relative to the first. We calculated the cross-correlation of the two configurations for various sizes of Eulerian grids. Shown in Fig. S5 is the success rate called the “Valid Detection Probability”^11,12^ (VDP) which identifies the likelihood that the cross-correlation is strongly peaked at the position of the imposed displacement. The VDP is plotted vs. the product of the number density of pairs of the same tracer from one frame to the next (normalized by the area of the grid element), $N_{i}$, the fraction of image pairs (from the same tracer) that exhibits displacement parallel to the field-of-view and remain in the same grid element, $f_{i}$, and the fraction of pairs that do not move away from the focal plane of the field-of-view, $f_{o}$. If the product $N_{i} f_{i} f_{o}$ is large enough, then VDP approaches unity. We show two calculations that illustrate how the VDP is affected by choice of grid size (Fig. S5a) and magnitude of the displacement (Fig. S5b). Our calculations are consistent with those in Ref.^11,12^ Taking as an example of a successful application of PIV, we estimate from the movie provided in Ref.^6^ there are 1600(100) tracers covering a 4 cm^2^ field-of-view from which a velocity vector field was obtained for a grid divided into ~ 36 by ~ 36 elements. Using these values, we estimate $N_{i} = 1.23\left( 8 \right)$ per grid element or 400(25) per cm^2^ and assuming $f_{i} = { }f_{o} = 1$, the product $N_{i} f_{i} f_{o}$ yields a VDP > 0.5. For our experiment, we observe ~ ${\mathcal{O}}\left( {10^{2} } \right)$ excimer events (tracers) in each frame covering the 1 cm^2^ area of the plane illuminated by the laser, i.e., $N_{i} \sim {\mathcal{O}}\left( {10^{2} } \right)$ per cm^2^. (The power of our laser is sufficient to excite all excimers in the illuminated plane so there are many more excited excimers, yet most do not emit photons that enter the camera’s lens.) Assuming a grid size equal to that of Ref.^6^ or 18 by 18 (for 1 cm^2^), and accounting for the probability of $\frac{1}{42}$ that the same excimer emits photons captured by the camera in sequential frames, then at most $N_{i} f_{i} f_{o} \sim {\mathcal{O}}\left( {10^{ - 2} } \right)$ per grid element, consequently, the VDP is near zero. For the conditions of our experiment, we do not expect *PIV to be successful when applied to fluorescence from individual excimers (i.e., transiently visible tracers)* and confirmed as much applying openPIV software^48^ to our data.

What the neutron capture approach lacks in terms of (localized) signal compared to other approaches is possibly offset by the opportunity to monitor flow simultaneously about large scale structures in three dimensions(A similar dichotomy is faced in the choice between using a nuclear reactor—a neutron source with high time averaged flux ideally suited to examine discrete regions of reciprocal space—or a pulsed neutron source—a source with high instantaneous brightness best suited to acquire data from an expansive and continuous range of reciprocal space—for neutron scattering). Rather than tracking the motion of individual excimers that are not consistently visible to the camera, we developed a means to quantify flow by observing the motion of ensembles (clouds or clusters) of excimers (because some excimer events in a cluster will be recorded by the camera). To observe flow requires means (1) to identify individual excimer clusters in an image and (2) to observe the motion of the clusters from one image to the next. To identify excimer clusters, we applied a machine learning algorithm to each image of excimer peaks with intensities > 4σ (e.g., those shown in Figs. S3 and S4). To track the clusters, we used a metric to estimate the likelihood that a cluster in one image is correlated (i.e., is the same) as a cluster in the subsequent image.

To facilitate automation, an unsupervised clustering algorithm is desirable. Further, to avoid biasing the number of clusters in any given image [because the intensity of the fluorescence can change with time, Fig. 2 (right)], an algorithm that does not constrain a priori the number of clusters is preferred. We found the Mean Shift clustering algorithm^49^ worked best. This approach identifies arbitrarily shaped objects as clusters and obtains the centroids of the clusters using a well-established pattern recognition procedure—the Mean Shift procedure^50,51^. During execution of the algorithm, a so-called mean-shift-vector is calculated that points in the direction of increasing density of events (to form the cluster). Upon convergence, i.e., no further significant increase of density detected^52^, the centroid of the cluster and the excimer peaks comprising the cluster are reported. The approach regards the image as a probability density function of a parameter—in our case the positions of fluorescence in the same frame. The Mean-Shift approach differs from another popular class of clustering approaches that use hierarchical clustering based on proximity measures^53^. A detailed description of the process to analyze the data and application of the unsupervised clustering formulism is given in the Supplemental Materials^15^.

**Figure 4:**
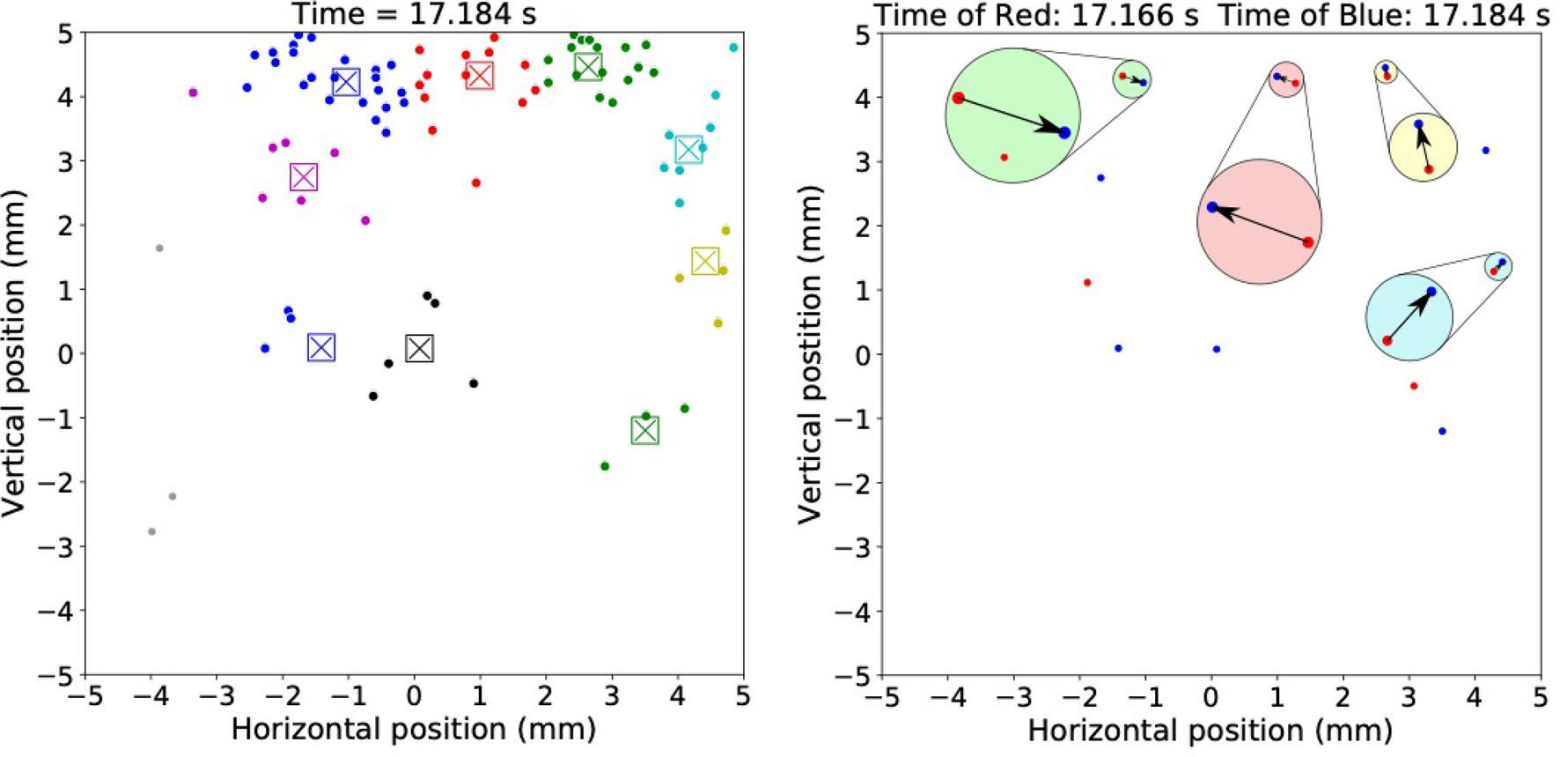
(left) Excimer fluorescence having intensity $> 4\sigma$ above background (small dots) color-coded according to cluster identity obtained using the MeanShift clustering algorithm (bottom heater power = 7.32 mW). The center of a large symbol represents the cluster centroid. (right) Positions of centroids for times of 17.184 (shown as the large symbols for this time on the left) and 17.166 s corresponding to blue and red dots, respectively. Specifically, the blue symbols in this figure (right) correspond to the positions of cluster centroids in this figure (left). In this figure (right), four pairs of red and blue centroids were identified by the correlator metric discussed in the text as being the same clusters that moved from time = 17.166 to time = 17.184 s (arrows). The arrows are the four displacement vectors calculated for the four cluster centroid pairs. The figure also shows pairs of displacement vectors magnified five times.

An example of the application of the algorithm to data collected for time slice of 17.184 s of Fig. 2 is shown in Fig. 4 (left). The large symbols represent the centroids obtained for the clusters; the small dots of the same color show excimers peaks comprising the same cluster. A movie of the clusters vs. time showing the upward motion of the clusters is presented in Fig. S6. The time-average number of clusters identified per frame from Fig. S2 was 6(2). The time-average number of excimer peaks in each cluster of Fig. S6 was 9(7). Gray dots in Figs. 4 (left) and S6 are excimer peaks (~ 15%) that do not belong to any cluster and were ignored in our analysis.

Next, we compare centroids of the *i*-th image taken at time $t_{i}$ with those in the next image at $t_{i + 1}$. The comparison involved summing over the distances between centroids in the first image with those in the second image for every combination of cluster pairs (a pair is a cluster in the first frame and ideally the same cluster in the next frame) that did not exceed a distance of $D$ apart. The approach is very similar to the so-called particle displacement determination algorithm^14^. Given the velocity of flow, there are values of $D$ that are not reasonable, i.e., would represent a velocity that far exceeds the mean velocity of the flow. The influence of choice of $D$ on the mean velocity computed from the excimer clusters is shown in S7. For the condition of 7.3 mW heater power, we chose $D = 390 \,{\mu m}$ (10 $\times$ the camera pixel dimension) so that the velocity of cluster-pairs on average equaled the mean velocity of the motion of fluorescence over the same field-of-view and time interval [Figs. 2 (red line) and 3]. Application of this metric (and the particle displacement algorithm) can yield erroneous results^14^. For example, the production of a new excimer cloud by neutron capture in one frame could be mistaken for motion of an excimer cloud from the previous frame. However, this situation cannot be realized for clouds that are tracked in the shadow of the neutron beam.

The movement of the centroid of the cluster from one frame to the next is the velocity projected onto the focal plane of the cluster. Shown in Fig. 4 (right) are the cluster-centroids (not the excimer peaks) for time = 17.166 s (red symbols) and time = 17.184 s (blue symbols). Note the blue symbols in Fig. 4 (right) correspond to the centers of the squares shown in Fig. 4 (left). Using the correlator algorithm, four cluster pairs Fig. 4 (right) were identified for images taken at 17.166 and 17.184 s. In Fig. 4 (right) an arrow shows the displacement of the centroid of one cluster in the first frame to the next. Centroids of clusters that are not connected by an arrow are separated by a distance greater than $D$, thus, such clusters do not form a cluster-pair—they are different clusters, not the same cluster that just moved. Fig. S8 shows the displacement vectors of correlated cluster-centroids from one frame to the next for a 1000 frames of one experiment (heater power 7.3 mW). Clusters and the cluster-centroids were identified with machine learning applied to the positions of the excimer peaks with intensity > 4 $\sigma$ shown in Fig. S4, and the result shown in Fig. S6. The correlator algorithm identifies cluster-centroid-pairs across sequential frames in Fig. S6, and vectors connecting the pair of centroids—the displacement vectors. The displacement vectors obtained from data in Fig. S6 are shown in Fig. S8. The motion of a cluster is obtained by dividing the displacement vector by the time between frames, 18 ms. The consistency between motion of the clusters (Fig. S8) and motion of the fluorescence (Fig. S2) demonstrates the opportunity of the approach to quantify the velocity vector field flow in two dimensions extracted from individual excimer clouds and sparse data.

**Figure 5:**
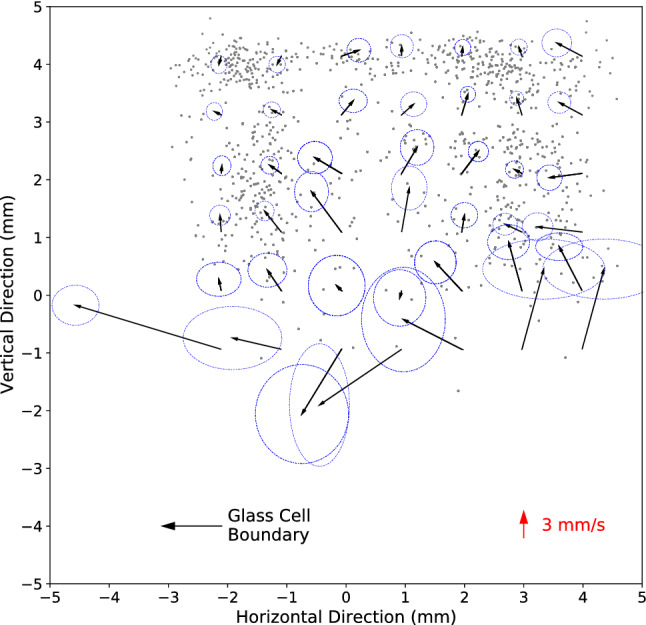
Box averaging of velocity vectors inferred from correlated-cluster-pairs (gray dots show locations of ~ 4800 cluster-centroid-pairs) from all images acquired at 7.3 mW heater power. An arrow indicates the mean velocity of correlated-cluster-pairs inside a 2 mm × 2 mm box (an example is the red box) centered at the origin of the arrow. The axes of the ellipse at the apex of the arrow represent the standard error on the mean for the two components of the velocity vector. The box was scanned in steps of 1 mm to produce the figure.

By superimposing all the correlated-cluster images for up to five experiments with the same heater power into a single image, we can obtain the velocity vector field of flow in the plane illuminated by the laser. The flow field was obtained by averaging the velocity vectors of cluster-pairs within a 2 mm by 2 mm box (red box, Fig. 5) and scanning the box across the field of view in 1 mm steps across a square lattice. The standard error on the mean of the horizontal and vertical components of the velocity are represented by the axes of the ellipse at the tip of the arrow. The gray dots show locations of correlated-cluster-pairs numbering 4841 for heater power of 7.3 mW. The standard error on the mean velocity is small where there are many cluster pairs. Away from the boundaries of the glass cuvette, the velocity vector flow field is primarily along the positive vertical axis corresponding to the direction of heat flux.

## Conclusion

In summary, we have demonstrated the ability to observe the motion of ${\text{He}}_{2}^{*}$ excimers through their fluorescence across a 1 cm^2^ field-of-view. We applied an unsupervised machine learning algorithm and a correlation algorithm to identify and track the centroids of individual excimer clusters in the focal plane of the camera as a function of time in a manner most closely resembling PTV. This allows us to obtain the velocity vector field of the flow from sparse data with a spatial resolution of ~ 39 ${\mu m}$ (the uncertainty in location of a centroid cluster), a temporal resolution of 18 ms across 1 cm in two dimensions. With additional lasers and cameras, the approach can be extended to observe flow in three dimensions. The density of excimer clusters can be increased by a factor of 10 by increasing the concentration of ^3^He accordingly. The increased density of ^3^He would be ~ 1% of the roton density and thus not expected to affect the hydrodynamics of He II at 1.7 K.^1^ With more excimer clusters and a faster camera, the spatial resolution will improve. Cameras for neutron imaging with Timepix3^54^ integrated chips record scintillation events with spatial resolution of 10’s of microns in event mode—meaning the absolute times of events are recorded continuously (with 1.6 ns precision). Use of event mode cameras would improve the reliability of the machine learning algorithm and the correlation algorithm for fluorescent imaging applications of ${\text{He}}_{2}^{*}$. For example, with continuous recording of event times, the value of *D* could be reduced increasing the confidence of the correlation algorithm—excimer clusters identified with machine learning could be tracked with the period of the laser (1 ms) rather than that of the PI-MAX3 camera (18 ms).

## Supplementary Information

Supplementary Figure 1.

Supplementary Video 2.

Supplementary Figure 3.

Supplementary Video 4.

Supplementary Figure 5.

Supplementary Video 6.

Supplementary Figure 7.

Supplementary Video 8.

Supplementary Information 9.

## Data Availability

The datasets generated and/or analyzed during the current study are available in the zenodo repository, https://doi.org/10.5281/zenodo.7051680. The codes used to analyze the data are available in the Github repository, https://github.com/mfitzsimmons44/Observing-Flow-with-He-II/.
