# Supplementary figures and images for: Observing flow of He II with unsupervised machine learning
Source: Sci Rep. 2022 Nov 27;12:20383. doi: 10.1038/s41598-022-21906-w (PMC9701805; doi:10.1038/s41598-022-21906-w)

# BN Neutron Blocker

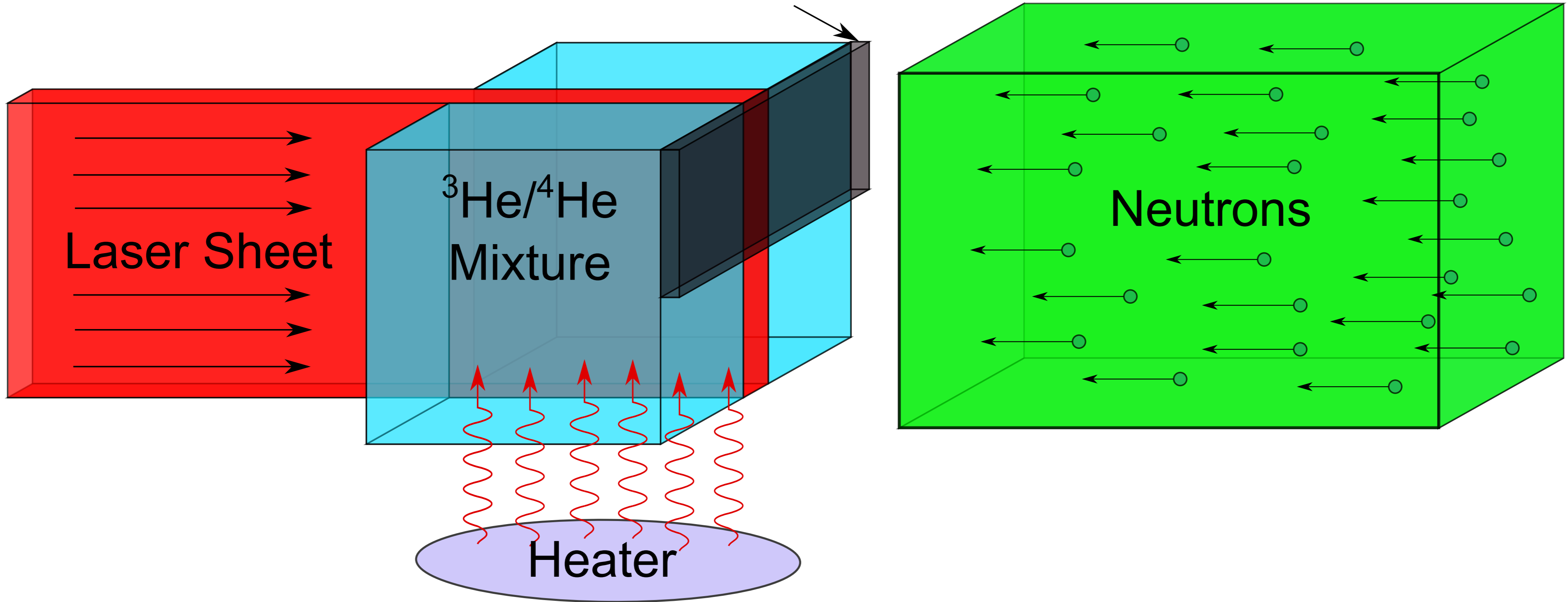

Supplement: Supplementary file 1 — Supplementary Figure 1. [file 41598_2022_21906_MOESM1_ESM.pdf]

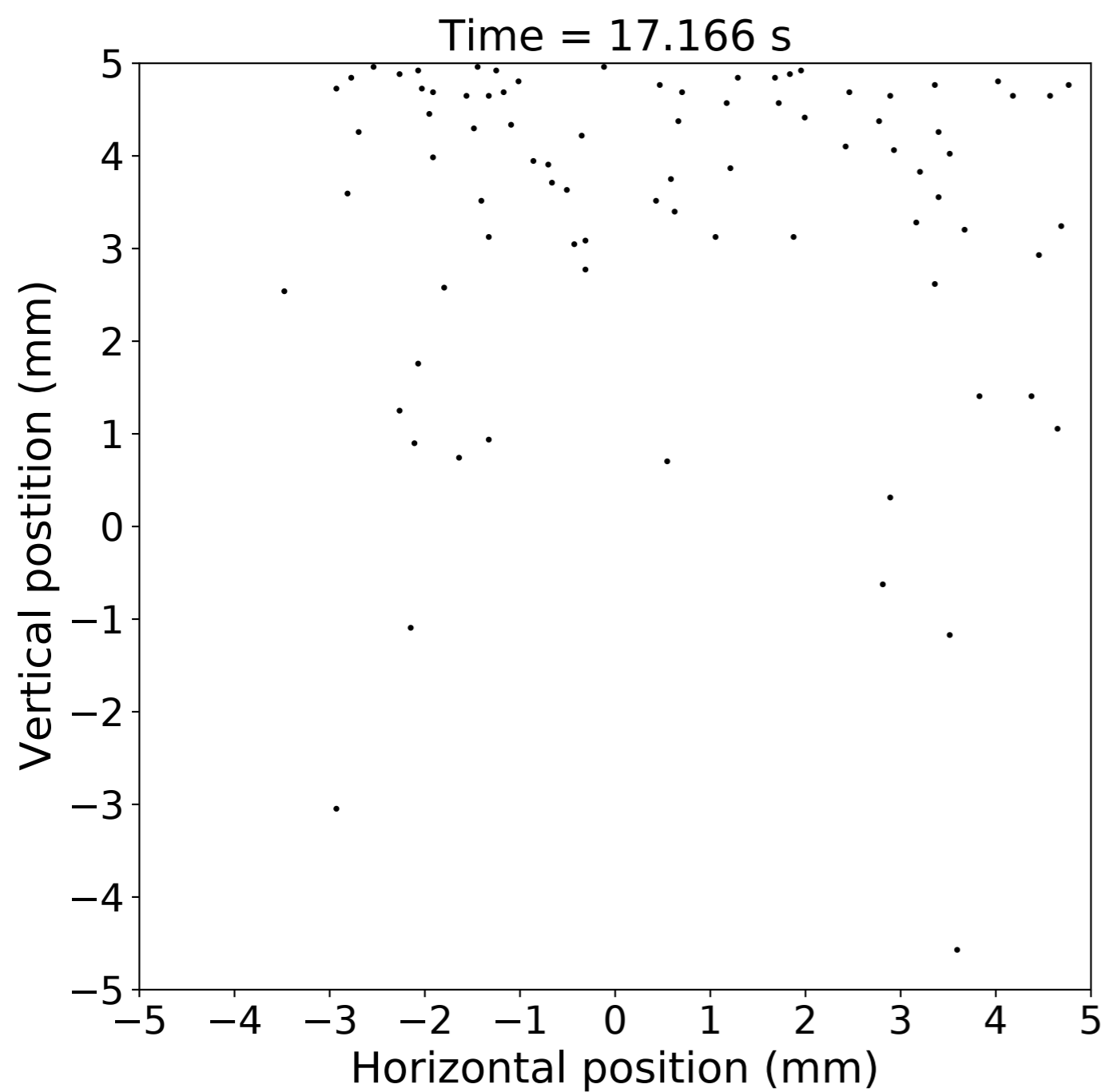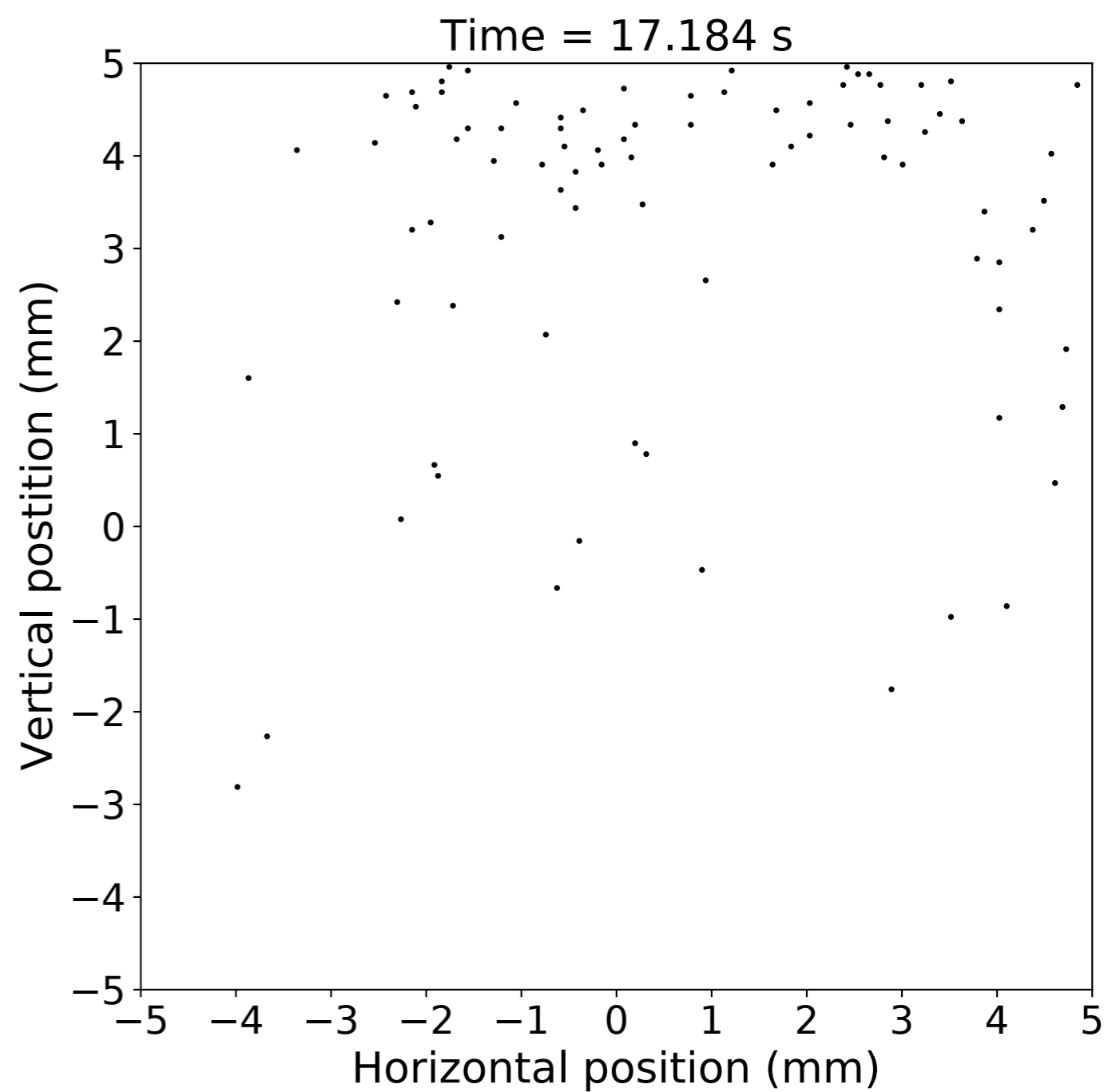

Supplement: Supplementary file 3 — Supplementary Figure 3. [file 41598_2022_21906_MOESM3_ESM.pdf]

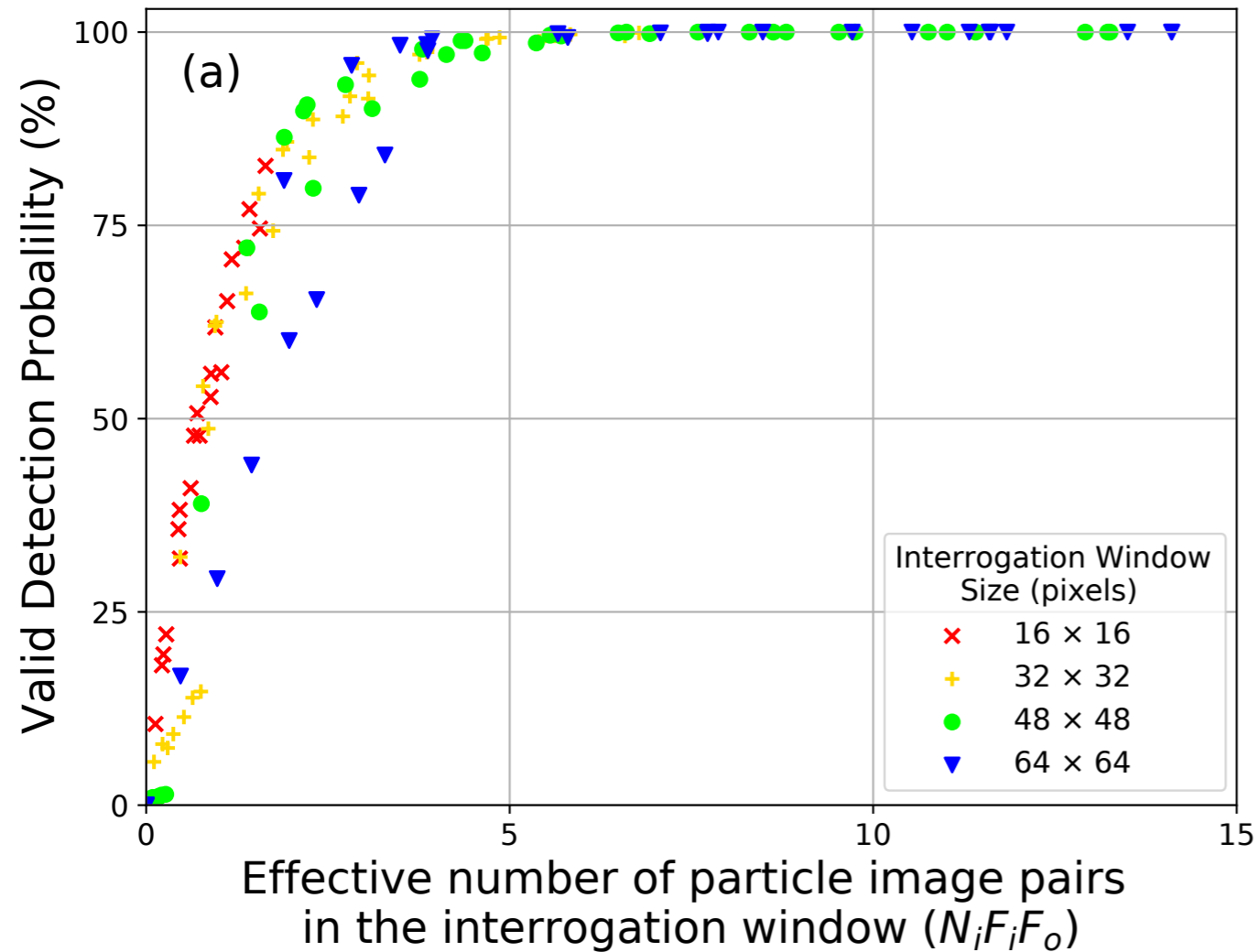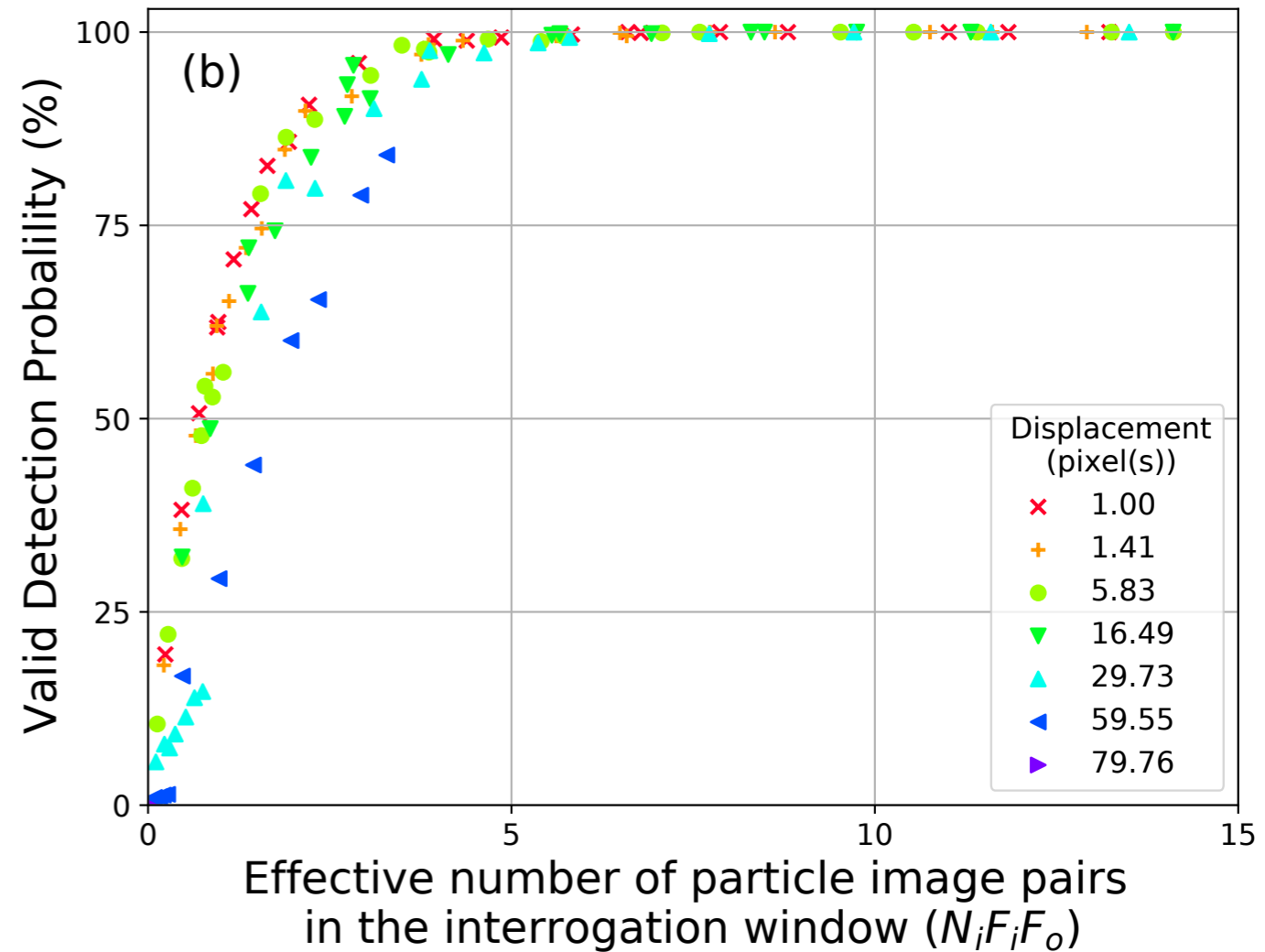

Supplement: Supplementary file 5 — Supplementary Figure 5. [file 41598_2022_21906_MOESM5_ESM.pdf]

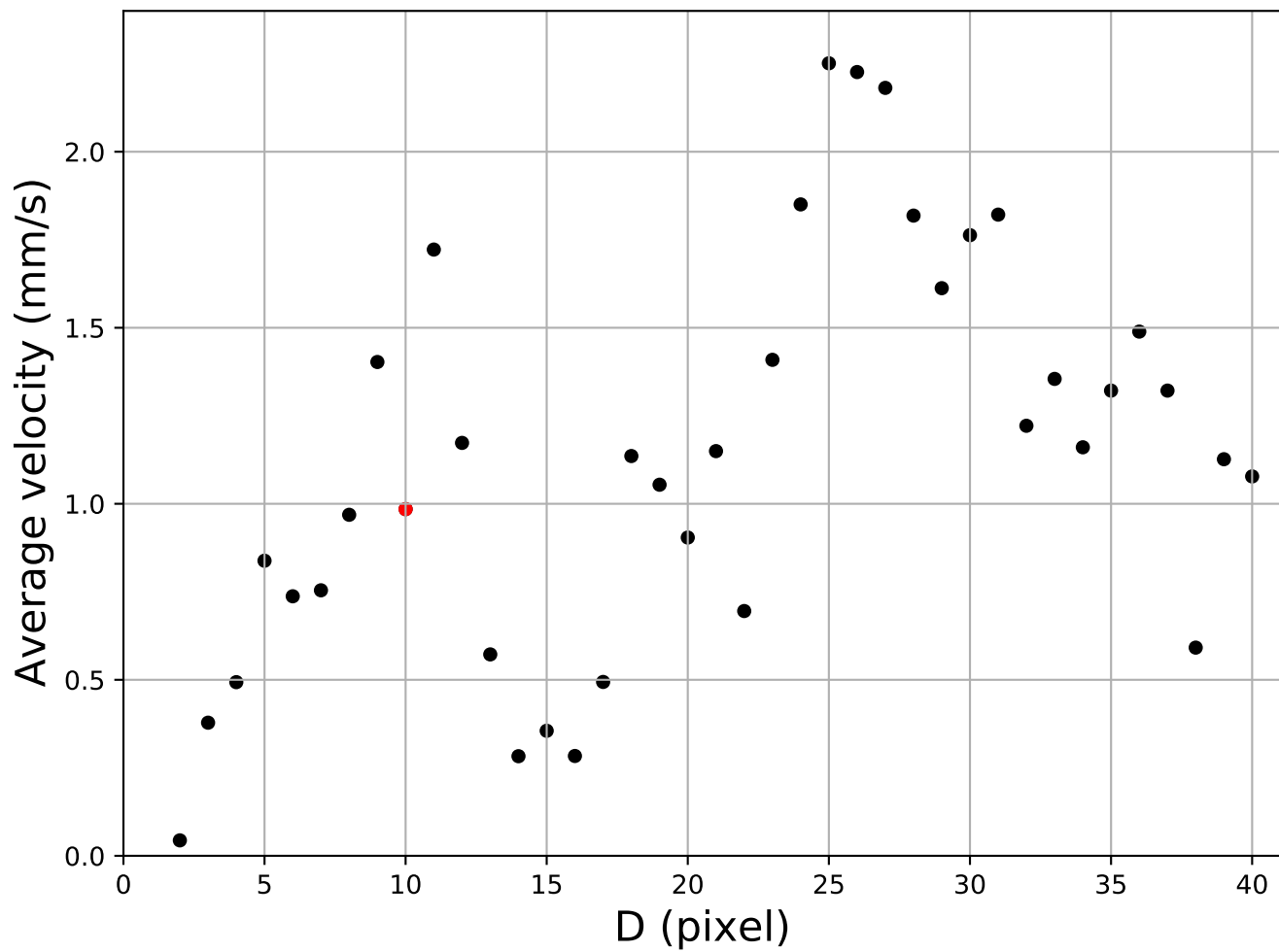

Supplement: Supplementary file 7 — Supplementary Figure 7. [file 41598_2022_21906_MOESM7_ESM.pdf]
