# Supplementary material for: Observing flow of He II with unsupervised machine learning
Source: Sci Rep. 2022 Nov 27;12:20383. doi: 10.1038/s41598-022-21906-w (PMC9701805; doi:10.1038/s41598-022-21906-w)
Supplement: Supplementary file 9 — Supplementary Information 9. [file 41598_2022_21906_MOESM9_ESM.pdf]

Supplementary Materials for

"Observing flow of He II with unsupervised machine learning"

X. Wen, L. McDonald, J. Pierce, W. Guo and M.R. Fitzsimmons*

*Corresponding author: M.R. Fitzsimmons mf3@ornl.gov

The supplementary material contains figures and movies that are a crucial component of our manuscript. We recommend using Adobe Acrobat to view this document or the movies may not play correctly. The movies can be played from this document and are also available in mp4 format.

Detailed discussion of data analysis

A typical experiment involved collecting two datasets. The first, called the "Raw Dataset", recorded ~1000 images at a rate of 55.6 Hz. For *Raw Dataset*, the three lasers were on, laser shutter open, neutron beamline shutter open and local (fast) neutron shutter open. The liquid ^3He:^4He mixture was condensed in the bulb as described in the paper. The second, called the "Background Dataset", recorded ~1000 images in the same manner as the *Raw Dataset* except the local neutron shutter was closed. This ensured no excimers were produced by the cold neutron beam, while contributions of electronic noise, cosmic and gamma ray interactions with the camera etc. to the data were the same for both measurements.

The signal in the *Background Dataset* was reasonably uniform equivalent to ~600 (arbitrary units) per pixel. Occasionally in the field of view some pixels (~2.6%) were persistently on, i.e., always had a very large signal. These pixels were flagged as defective (camera) pixels and were not used in our analysis. From the remaining pixels we calculated the mean signal per frame, $\bar{B}$, and the root-mean-square of the distribution, σ.

Next, we calculated the net signal above background which is the difference between *Raw Dataset* and $\bar{B}$. We call the result "Net Dataset". In Fig. S2 (and Fig_S2.mp4), we show a movie of *Net Dataset* (heater power = 7.3 mW) integrated over a 0.5 s time window. N.b., the integration serves only to provide visual clarity for the movie in Fig. S2 (and Fig_S2.mp4); the integration was not used in analysis of the data.

As described in the main text, using *Net Dataset* and the peak finding python routine: `photutils.detection.`**`find_peaks`**, we located all peaks in *Net Dataset* with signals > 4σ. Our call to this routine:

```
photutils.detection.find_peaks(array, threshold = dthreshold,
box_size = 5)
```

with dthreshold = $4\sigma = 4 \times 15$.

This result produced a list of excimer peaks as a function of position across the field of view and as a function of frame number, which is a proxy for time. An example for one pair of frames for heater power = 7.3 mW is shown in Fig. S3. A movie of these events (heater power = 7.3 mW) for every frame is shown in Fig. S4 (and Fig_S4.mp4).

We applied an unsupervised machine learning algorithm optimized to identify clustering of excimer peaks (> 4σ in *Net Dataset*) and to find the locations of the centroids of the clusters for each frame. We investigated numerous algorithms and chose the so-called "Mean-Shift" algorithm[1] for reasons discussed in the text as being most suitable for our experiment. The call to the routine was:

```
sklearn.cluster.MeanShift(*, bandwidth=None, seeds=None,
bin_seeding=True, min_bin_freq=1, cluster_all=True, n_jobs=None,
max_iter=300)
```

The default *bandwidth=None* forces a call to another routine: *sklearn.cluster.estimate* that estimates the bandwidth to be used by *sklearn.cluster.MeanShift.* The choice of *bandwidth* is related to the size of the cluster. This is a number produced by *sklearn.cluster.estimate*.[2] The call to the routine was:

`sklearn.cluster.`**`estimate_bandwidth`**`(X, *, quantile=0.1, n_samples=None, random_state=0, n_jobs=None)`

As input to this routine the user provides a value of *quantile*, which for our case is related to the distance between pairs of excimer peaks. The choice of *quantile* affects the value of *bandwidth* and ultimately the size of the cluster and thus number of clusters. For example, in some images we see many excimer peaks, so we expect more clusters to be identified in such an image than had there been very few peaks. We used a value of *quantile = 0.1* to achieve cluster sizes that contained a number of excimer peaks that was consistent with the number we expect (and discussed in the main text) based on the energy released during neutron absorption, the path length of the ionizing radiation and the frequency of neutron absorption. The value of `bin_seeding = True` provided a reasonably uniform distribution of clusters. The same call to `sklearn.cluster.`**`MeanShift`** was used for analysis of all experiments. A movie showing the color-coded clusters for every frame (heater power = 7.3 mW) is shown in Fig. S6 (and Fig_S6.mp4). Excimer events in a common cluster are shown as small dots of one color with the large square of the same color showing the location of the cluster centroid. A list of the positions of the centroids for every frame is produced using the Jupyter Notebook code[3] and data in the data repository.[4] About 85% of all peaks are mapped to clusters. The remaining are too dispersed and too far from clusters to be associated with a cluster.

With the cluster centroids determined for each time step (frame), a metric to identify highly correlated centroids in adjacent frames was applied. In other words, the metric identified a centroid in one frame that moved after 18 ms and is seen at a different position, though within *D* of the first position (see main text), in the next frame. As described in the main text and references therein, the metric is one used in other applications of PTV. The calculations produce displacement vectors

connecting one cluster-centroid with what is expected to be the centroid of the same cluster in the next frame. The displacement vectors identified by the correlator metric for every pair of frames (heater power = 7.3 mW) is shown in Fig. S8 (and Fig_S8.mp4). Using the frequency of the camera recording, and the displacement vectors, the velocity vector of a centroid is calculated.

The last step in our analysis is to calculate the velocity vector flow field using an Eulerian grid applied to the displacement vectors. The flow field represents the position dependence of the velocity vector integrated over all time. To make this calculation, we superimposed displacement vectors for all pairs of frames for all experiments that used the same heater power. Up to five experiments using the same heater power were combined to form the velocity flow field. To obtain the flow field, we constructed an Eulerian grid consisting of equal spaced partitions of the field of view along the horizontal and vertical directions such that the lattice of the grid had a dimension of 1 mm. At the center of each grid point we calculated the mean velocity vector using every displacement vector within a box of 2 mm by 2 mm and the standard error of the mean (of the velocity vectors within the box). This process produced Fig. 5 (heater power = 7.3 mW) in the main text. The result shows upward motion of the flow consistent with the motion of the fluorescence observed in the movie Fig. S2 (and Fig_S2.mp4).

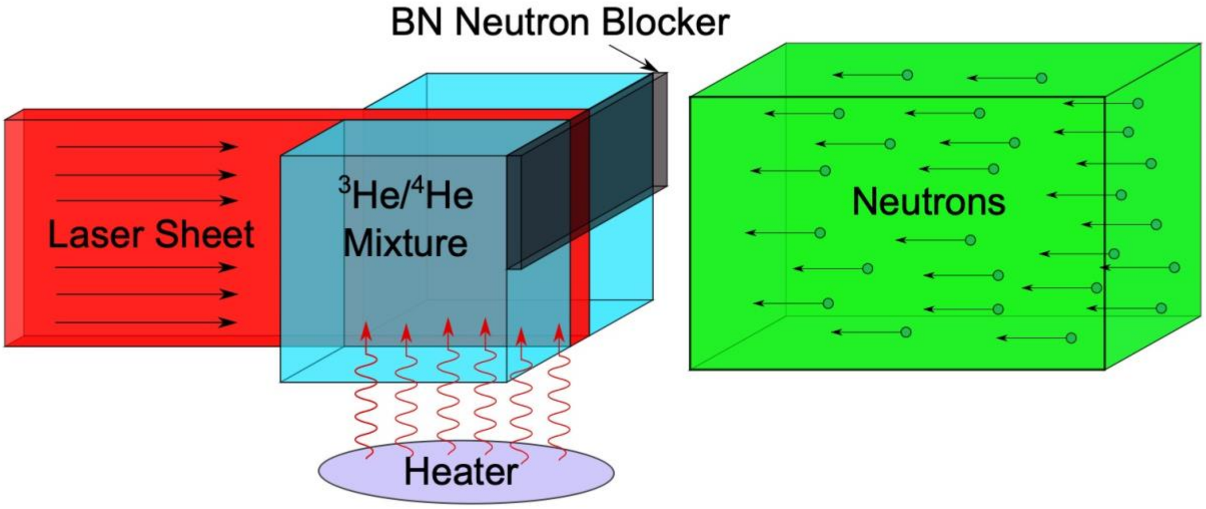

Fig. S1 A laser sheet (red) ~10 mm high by 1mm thick illuminated a quartz cell of liquid ^{3}He / ^{4}He mixture (cyan) from the left. A collimated neutron beam (green) ~12mm high by 8mm thick consisting of wavelengths ranging from 2.8 Å to 5.8 Å irradiate the liquid from the right. The focal plane of the camera lies within the laser sheet, and the field of view was adjusted to be 1cm by 1cm. A neutron blocker (gray) made of Boron Nitride (BN) shadows the upper half of the liquid mixture so that He_2^* excimers are only created in lower half of the cell. He_2^* excimers. A heater (purple) installed below the field view is the source of heat that causes flow of normal component upwards. The excimers track the motion of the normal component flow and so drift upwards. Only excimers illuminated by the laser can fluoresce.

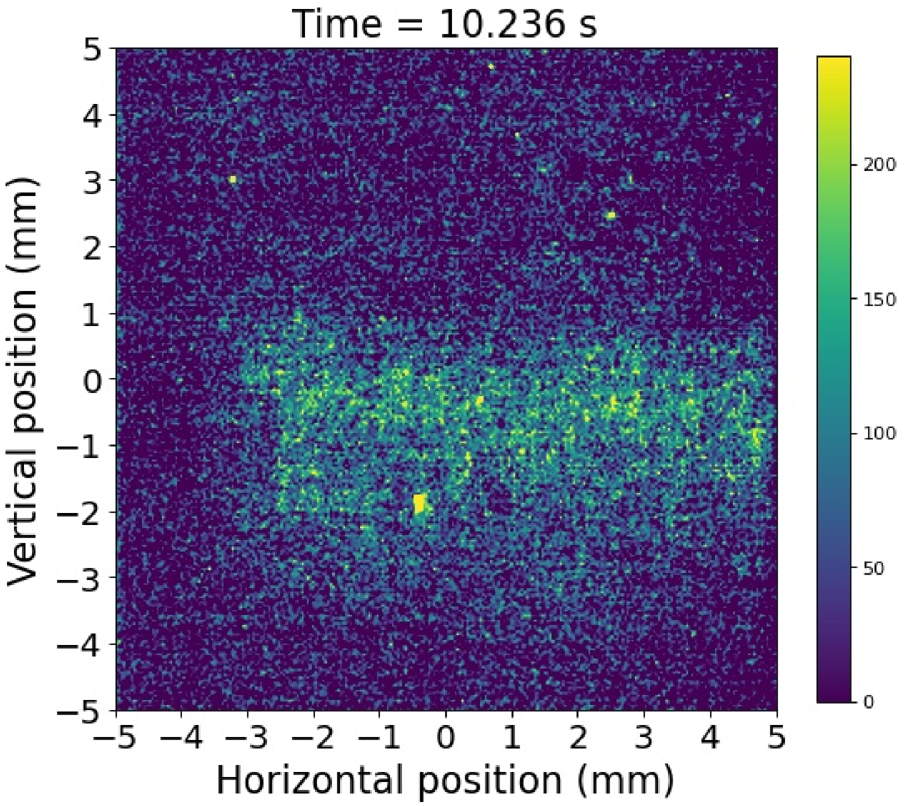

Fig. S2 A movie of the fluorescence recorded by the camera's field of view as a function of time. The background noise has been subtracted. The neutron shutter was opened at 5 s, the laser shutter opened at $t_l = 10$ s, and the bottom heater energized at $t_h = 12$ s. All excimers are first seen in the region where the vertical position is < 0 because no neutrons enter the vertical region > 0. After the bottom heater was energized (7.3 mW), the thermal gradient caused the normal component of He II to move upwards. The vertical shadow near the center of field of view is the shadow of our sample rod that holds the heaters. The color bar scale represents the net signal (above background) of a pixel recorded by the camera.

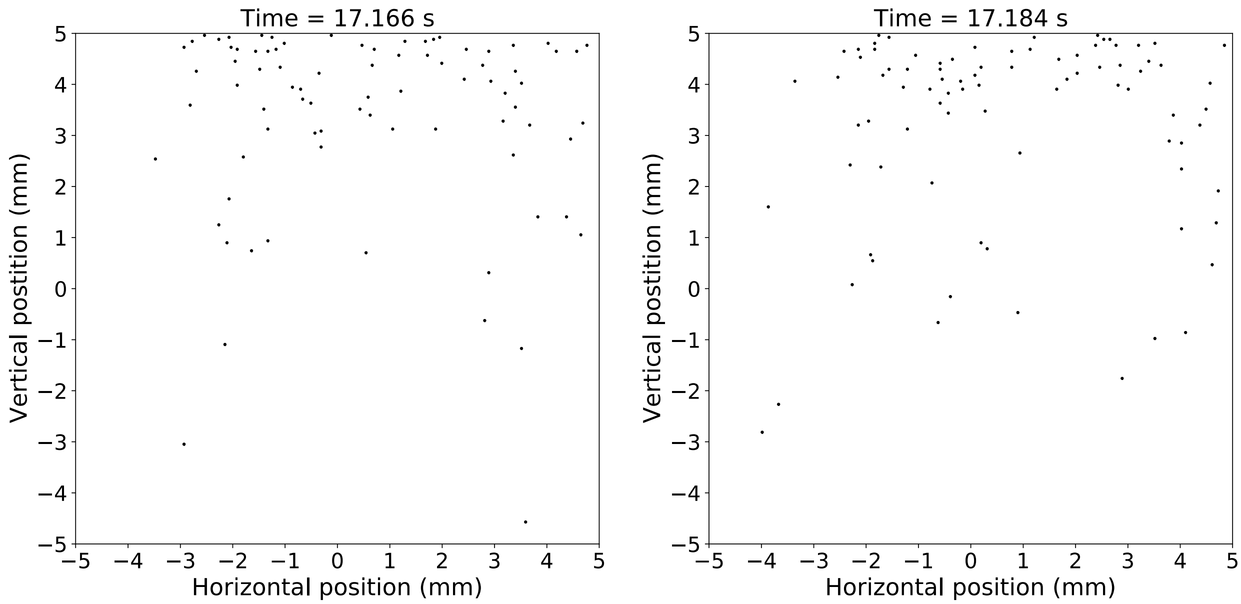

Fig. S3 Images of events identified in two sequential frames (heater power 7.3 mW). Each frame contains ~80 events (or records of 80 photons) over the 1 cm^2 view. These events all have intensity greater than 4-sigma above the background. Given that the trajectory of an emitted photon is random, an excimer that produce a photon captured in Fig. S3 (left) has only a 1 in 42 chance to produce a photon in the second frame, Fig. S3 (right).

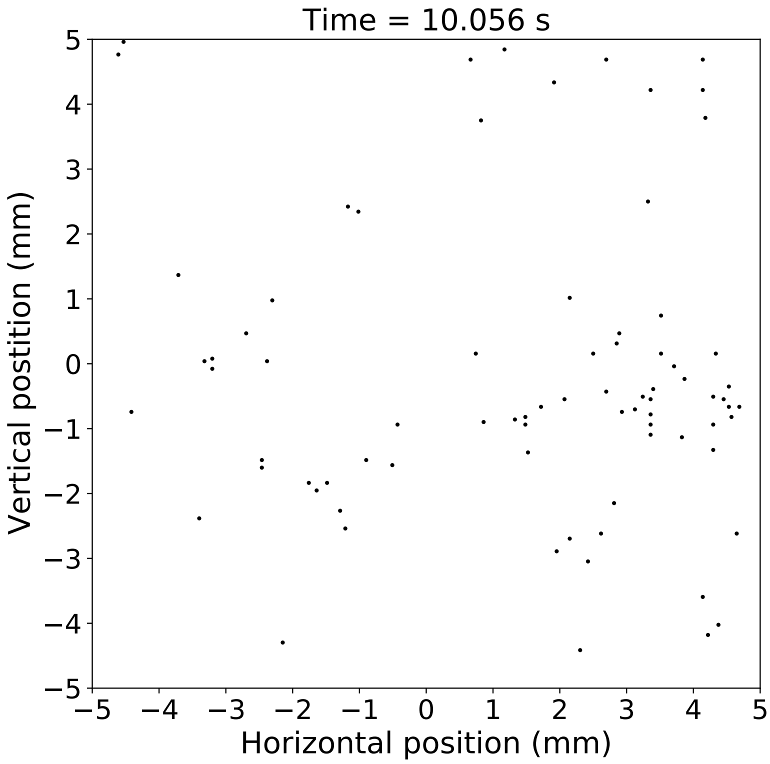

Fig. S4 A movie showing the locations of the excimer peaks having intensity of > 4σ (four times the root-mean-square of the background measurement). The two frames shown in Fig. S3 are part of this movie. The local (fast) neutron shutter was opened at time = 5 s. The movie begins at time = 8 s. The laser shutter was opened at time = 10 s. The heater was energized at time = 12 s (power of 7.3 mW).

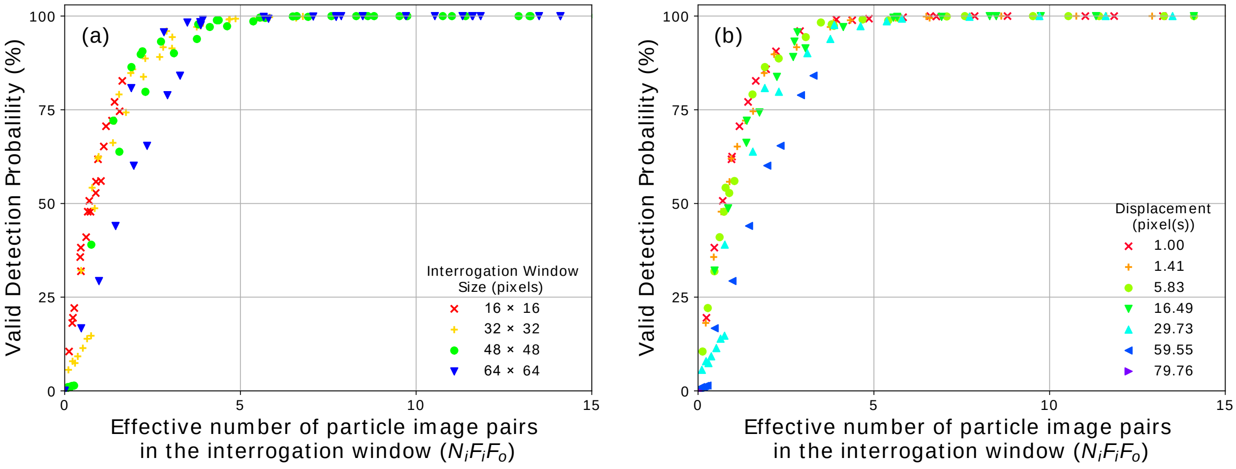

Fig. S5 A simple simulation results of Valid Detection Probability (VDP) vs. the effective number of particle-image-pairs in the interrogation window using a cross-correlation function. Fig. S5 (a) shows the effect of interrogation window size on the VDP, and Fig. S5 (b) shows the VDP with different imposed displacements of the tracer particles. The reliability of cross-correlation scales with the "Valid Detection Probability" (VDP). Even with the best choice of interrogation window size and the smallest tracer displacement, the effective number of particles image pairs $N_i f_i f_o$ should be > 3. For our experiment, if we regard individual excimer events, the effective number of image pairs is only $N_i f_i f_o \sim \mathcal{O}(10^{-2})$ per grid element. Thus, we cannot apply PIV and cross-correlation to individual excimers. Instead, we are motivated to apply PTV techniques to track flow represented by motion of excimer *clusters*.

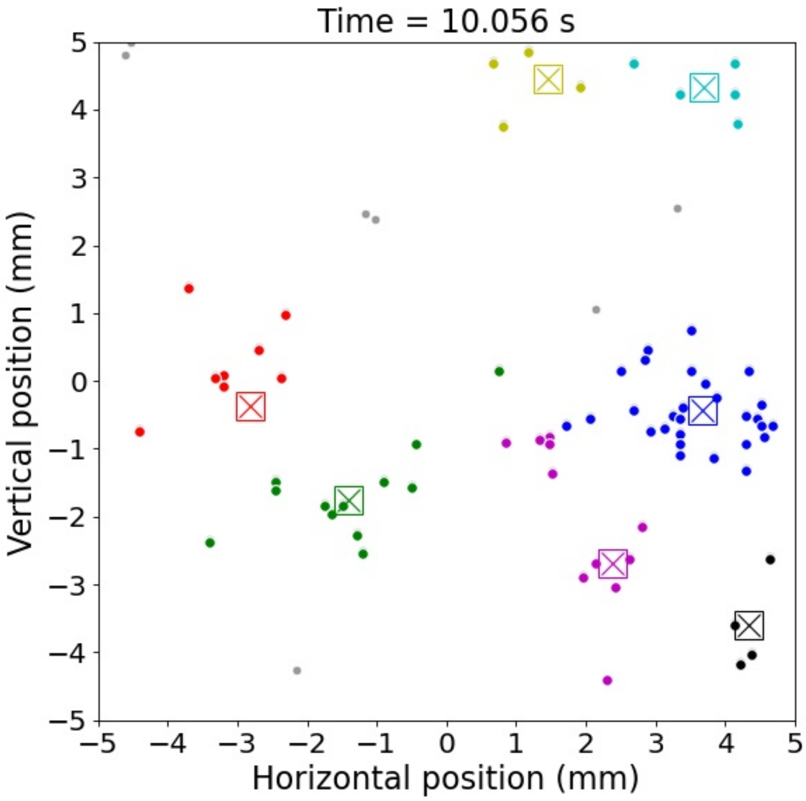

Fig. S6 A movie of the clusters vs. time showing upward motion of the clusters for the condition of the bottom heater being energized at 7.3 mW. The center of the large symbols represents the centroids of the clusters, and the small circles are the fluorescence events recorded by the camera and identified as comprising a cluster Mean Shift algorithm. A typical cluster contains $\sim\mathcal{O}(10^{1-2})$ events. The small colored circles appear at the same positions and times as shown in Fig. S4. Gray dots indicate events (orphans) that were not associated with a cluster.

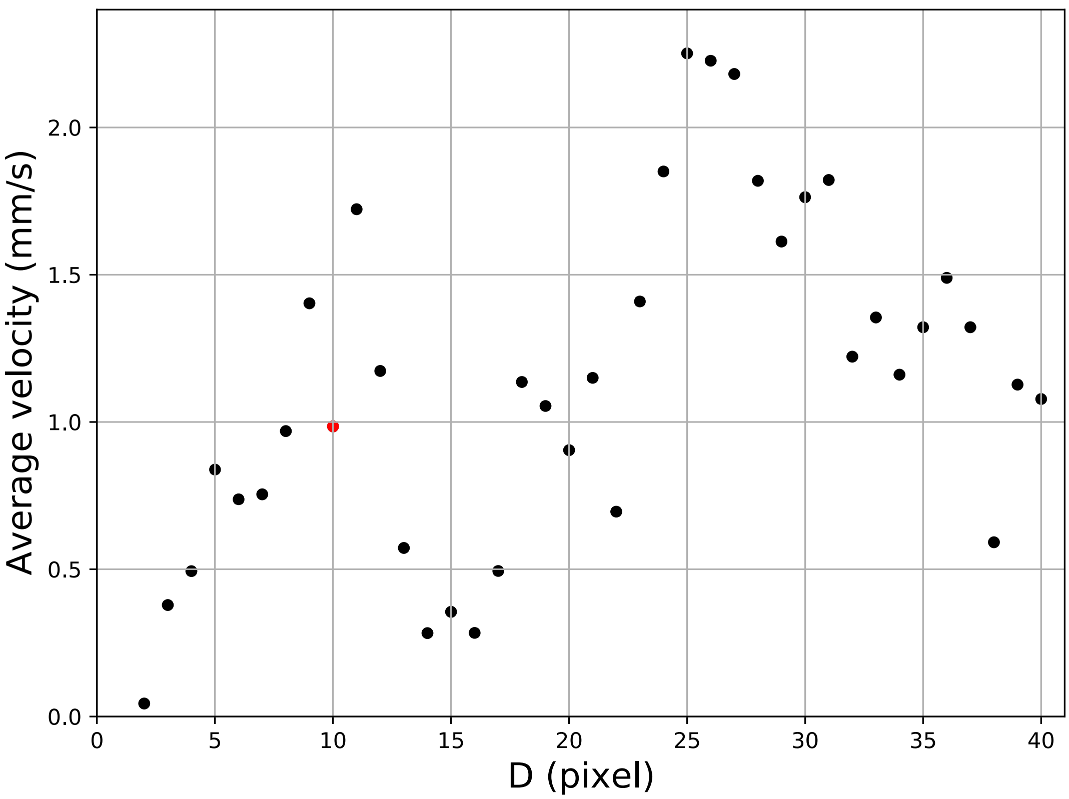

Fig. S7 The influence of choice of D on the correlator metric. The vertical axis shows the average velocity of all the image pairs identified with our metric when choosing D as a search radius limit. With 7.3 mW heater power applied, we can directly measure the average velocity to be 0.985 mm/s from the motion of the excimer fluorescence (shown in Fig. 2). We choose D = 10 pixels ($390\ \mu m$) to achieve a similar average velocity of 1 mm/s when integrated over the same field-of-view and same range of time used to obtain the mean velocity from the motion of the fluorescence. The value of D is proportional to the mean velocity. Using D = 10 pixels for 7.3 mW data and the slope of the blue line in Fig. 3, we obtain D = 377 μm for 3.69 mW, D = 418 μm for 14.69 mW, D = $445\ \mu m$ for 22 mW and D = $472\ \mu m$ for 29.41 mW data.

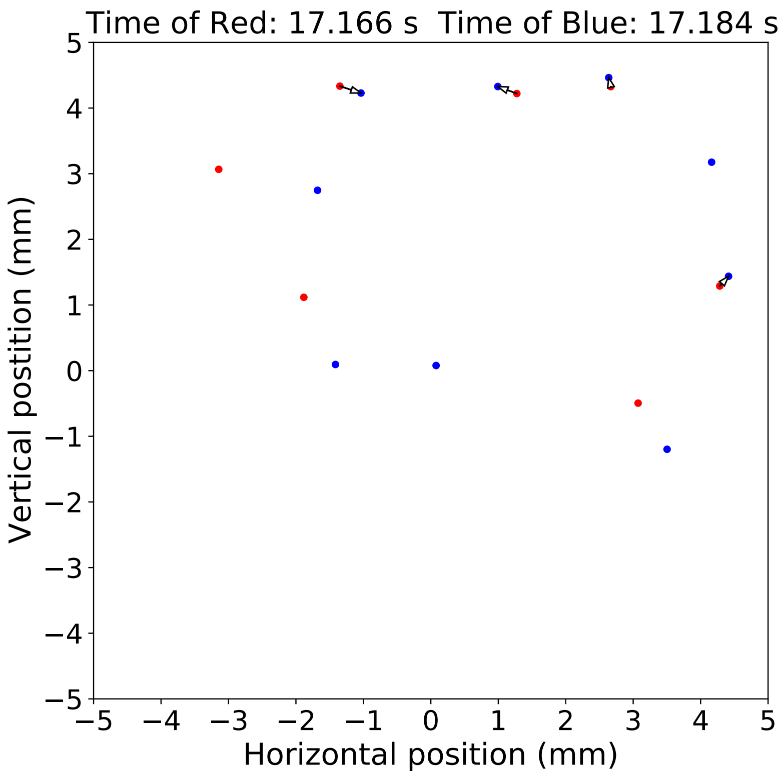

Fig. S8 Motion of the excimer clusters centroids. The red dots represent the cluster centroids of the first frame of an image pair, and the blue dots represent centroids in the second frame. If the distance between a red dot and a blue dot is $\leq D$, the pair are identified by the correlator metric as the same centroid in the two frames. The displacement vector divided by the time between adjacent frames (18 ms) yields the velocity vector for the cluster. The velocity vectors for thousands of such correlated cluster-centroid-pairs are used to produce the velocity vector flow field (Fig. 5, main text).

---

[1] https://scikit-learn.org/stable/modules/generated/sklearn.cluster.MeanShift.html

[2] https://scikit-learn.org/stable/modules/generated/sklearn.cluster.estimate_bandwidth.html

[3] https://github.com/mfitzsimmons44/Observing-Flow-with-He-II/

[4] https://doi.org/10.5281/zenodo.7051680
